# Ceft-to-Ceft Study: Real-Life Experience with Ceftaroline and Ceftobiprole in Treatment of the Principal Infectious Syndromes in a Spanish Multicenter Hospital Cohort

**DOI:** 10.3390/antibiotics12121692

**Published:** 2023-12-02

**Authors:** Daniel Arnés García, Inés Pitto-Robles, Jorge Calderón Parra, Marina Calvo Salvador, Carmen Herrero Rodríguez, Laura Gisbert, Carmen Hidalgo-Tenorio

**Affiliations:** 1 Servicio de Medicina Interna, Hospital Universitario Virgen de las Nieves, 18014 Granada, Spain; darnes.garcia.sspa@juntadeandalucia.es (D.A.G.); ipitto.rodriguez.sspa@juntadeandalucia.es (I.P.-R.); 2 Unidad Enfermedades Infecciosas, Hospital Puerta de Hierro de Majadahonda, 28222 Madrid, Spain; jparra@salud.madrid.org; 3 Servicio de Farmacia, Hospital Puerta de Hierro de Majadahonda, 28222 Madrid, Spain; mcalvos@salud.madrid.org; 4 Unidad de Enfermedades Infecciosas y Microbiología, Complejo Hospitalario de Jaén, 23007 Jaén, Spain; carmen.herrero.sspa@juntadeandalucia.es; 5 Unidad de Enfermedades Infecciosas, Hospital Universitario Mútua de Terrassa, 08221 Barcelona, Spain; lgisbert@mutuaterrassa.cat; 6 Unidad de Enfermedades Infecciosas, Hospital Universitario Virgen de las Nieves, Instituto de Investigación Biosanitario de Granada (IBS-Granada), 18014 Granada, Spain

**Keywords:** ceftaroline fosamil, ceftobiprole medocaril, endocarditis, mortality, OPAT

## Abstract

Background: To compare the real-life effectiveness and safety of ceftaroline fosamil (ceftaroline-F) and ceftobiprole medocaril (ceftobiprole-M) for infections in hospitalized patients. Methods: This comparative, observational, retrospective, and multicenter Spanish study included patients receiving outpatient parenteral antimicrobial therapy (OPAT) and hospitalized patients treated for at least 48 h with ceftaroline-F or ceftobiprole-M between their first incorporation in the clinical protocol of each hospital and 31 July 2022. Results: Ceftaroline-F was administered to 227 patients and ceftobiprole-M to 212. In comparison to the latter, ceftaroline-F-treated participants were younger (63.02 vs. 66.40 years, OR 1.1; 95%CI: 1.001–1.05) and had higher rates of septic shock (OR 0.27; 95%CI: 0.09–0.81) and higher frequencies of targeted (57.7 vs. 29.7%; OR: 0.35; 95%CI: 0.18–0.69) and combined (89.0 vs. 45.8%, OR: 0.13; 95%CI: 0.06–0.28) therapies that were second line or more (82.4% vs. 64.6%%; OR 0.35; 95%CI: 0.18–0.69), and higher rates of infections due to Gram-positive cocci (92.7 vs. 64.7%, *p* = 0.001), bacteremia (51.9 vs. 21.7%, *p* = 0.001), infective endocarditis (24.2 vs. 2.4%, *p* = 0.0001), and mechanical ventilation-associated pneumonia (8.8 vs. 2.4%, *p* = 0.0001). Ceftobiprole-M was more frequently administered against polymicrobial infections (38.1 vs. 14.0%, *p* = 0.001), those produced by Gram-negative bacilli (19.7 vs. 6.0%, *p* = 0.0001), nosocomial pneumonia (33 vs. 10.6%, *p* = 0.0001), and skin and soft-tissue infections (25.4 vs. 10.1%, *p* = 0.0001). Patients treated with ceftaroline-F had a longer hospital stay (36 (IQR: 19–60) vs. 19.50 (IQR: 12–30.75, *p* = 0.0001) days), with no difference in infection-related mortality at 14 (13.2 vs. 8.0%, *p* = 0.078) or 28 (4.8 vs. 3.3%, *p* = 0.415) days or in dropout rate for adverse effects (2.2 vs. 0.9%; *p* = 1). Conclusions: The fifth-generation cephalosporins, ceftaroline-F and ceftobiprole-M, are safe and effective in real life, with no difference between them in health outcomes.

## 1. Introduction

The increase in antimicrobial resistance is one of the main public health threats worldwide, including in Europe and Spain [1]. The rise of multiresistant microorganisms is associated with higher health costs, more frequent antibiotic treatment failures, quality of life impairment, and increased mortality [2]. The latest World Health Organization report on antimicrobial resistance in 2020 described a 16.6% prevalence of carbapenem-resistant *Pseudomonas aeruginosa* and a 23.3% prevalence of methicillin-resistant *Staphylococcus aureus* (MRSA) in Spain, with the latter being above the European mean of 16.7% [3]. These data underscore the need for a carbapenem-sparing strategy in our setting, using bactericides other than carbapenems in patients with sepsis or septic shock, such as fifth-generation cephalosporins: ceftaroline-F and ceftobiprole-M.

Ceftaroline-F exerts high bactericide activity against Gram-positive cocci (GPC) such as *Streptococcus pneumoniae* (including third-generation cephalosporin-resistant species), beta-hemolytic *Streptococci*, and *Staphylococcus aureus* (SA), including MRSA [4]. Its spectrum against Gram-negative bacilli (GNB) is comparable to that of third-generation cephalosporins and includes *Hemophilus influenzae*, *Moraxella catarrhalis*, and most Enterobacteriaceae [5]. The majority of nonfermenting GNBs are resistant, including *Pseudomonas aeruginosa* [5,6]. The European Agency has approved ceftaroline-F for community-acquired pneumonia (CAP) and acute bacterial skin and soft-tissue infections (ABSSTIs) [7,8]. Given its pharmacokinetics and pharmacodynamics, it may be a useful alternative option against bacteremia, infective endocarditis (IE), nosocomial pneumonia (NP), and osteoarticular infections [9,10], especially in cases of therapeutic failure or resistance to other antibiotics, such as vancomycin or daptomycin [11]. 

Ceftobiprole-M exerts bactericide activity against GPC (SA, including MRSA, *coagulase-negative Staphylococci* (CoNS), *Streptococci* spp., and ampicillin-susceptible *Enterococcus faecalis* and *E. faecium*), and Gram-negative bacteria (GNB), including *Hemophilus influenzae*, *Moraxella catarrhalis*, and *Pseudomonas aeruginosa* [12]. It is, therefore, suitable for treating bacteremia, IE, ABSSTIs, and complicated urinary infections [13]. Experimental and animal models of staphylococcal infections in biofilms found their bactericide capacity to be higher than linezolid, vancomycin, and daptomycin [14,15]. These properties make them especially useful against infections associated with devices, endoprostheses, osteosynthesis material, or prosthetic valves. However, they are only authorized for CAP and NP in Europe [16,17], despite demonstrating their non-inferiority against ABSSTIs in comparison to comparators [18]. 

Clinical trials are evidently the gold standard for the approval and commercialization of novel pharmaceutical compounds; however, extrapolation of their outcomes is limited by the strict inclusion criteria and special conditions imposed [19]. The aim of real-life studies (Real World Data (RWD)) is to bridge the knowledge gap between clinical trials and clinical practice. This type of study has served to reduce medical costs, improve outcomes, and accelerate the incorporation of novel therapies and technologies into routine clinical practice [20].

Given this background and the limited scientific evidence available on fifth-generation cephalosporins, the objective of this study was to compare real-life effectiveness and safety between ceftaroline-F and ceftobiprole-M in the treatment of the principal infectious syndromes in hospitalized patients in Spain.

## 2. Results

### 2.1. Cohort Description 

This study included 227 patients treated with ceftaroline-F and 212 treated with ceftobiprole-M. In bivariate analyses, patients receiving ceftaroline-F were younger (63.02 vs. 66.4 years, *p* = 0.015), more frequently had cardiovascular risk factors (74 vs. 49.1%, *p* = 0.001), cardiovascular disease (atrial fibrillation (16.3 vs. 8.5% *p* = 0.014), and moderate–severe heart valve disease (18.9 vs. 4.2%, *p* = 0.001)) and were more frequently solid organ transplant recipients (10.1 vs. 2.4%; *p* = 0.001), were in the Intensive Care Unit (ICU) (20.3 vs. 4.7%; *p* = 0.001) or post-surgery unit (8.5 vs. 0.0%, *p* = 0.0001), and were in septic shock (21.1 vs. 4.2%, *p* = 0.001). Patients administered ceftobiprole-M had a significantly higher rate of solid organ neoplasm under active treatment (7.1 vs. 2.6%, *p* = 0.030) and sepsis (16.7 vs. 25%; *p* = 0.033) and more frequently received outpatient parenteral antimicrobial therapy (OPAT) (3.8 vs. 0.0%, *p* = 0.003). In multivariate analysis, ceftaroline-treated patients were younger (63.02 vs. 66.40 years, OR 1.1; 95%CI: 1.001–1.05) and had higher rates of septic shock (OR 0.27; 95%CI: 0.09–0.81) and higher frequencies of targeted (57.7 vs. 29.7%; OR: 0.35; 95%CI: 0.18–0.69) and combined (89.0 vs. 45.8%, OR: 0.13; 95%CI: 0.06–0.28) therapies that were second line or more (82.4% vs. 64.6%%; OR 0.35; 95%CI: 0.18–0.69). Table 1 lists the results for the remaining study variables. 

Ceftaroline-F was prescribed more frequently for bacteremia (51.9 vs. 21.7%; *p* = 0.001), IE (24.2 vs. 2.4%; *p* = 0.001), IMVAP (8.8 vs. 2.4%; *p* = 0.001), and fever without focus (3.5 vs. 0%, *p* = 0.003) and less frequently for respiratory infections (45.4 vs. 66%, *p* = 0.006), NP (10.6 vs. 33%; *p* = 0.001), and ABSSTIs (10.1 vs. 25.4%, *p* = 0.0001) (Table 2).

Patients treated with ceftaroline-F received a higher drug concentration (11.7 g (IQR 6.6–19.8 g) vs. 11 g (IQR 6.25–15 g, *p* = 0.015)) for a longer time (8 days (IQR 5–14 day) vs. 7 days (IQR 5–10 days, *p* = 0.023)), and it was more frequently administered in combination (89 vs. 45.8%, *p* = 0.001) as a targeted therapy (57.7 vs. 29.7%, *p* = 0.001) and as a rescue therapy after the failure of another antibiotic (82.4 vs. 64.6%, *p* = 0.001) (Table 1). Ceftaroline-F was most frequently combined with daptomycin (36.4%), meropenem (20.8%), and metronidazole (20.8%) (Supplementary Table S1), and the antibiotics most often received before ceftaroline-F were daptomycin (33.7%), meropenem (27.9%), and linezolid (20%) (Supplementary Table S2). 

### 2.2. Microbiological Isolation

Among patients treated with ceftaroline-F, microorganisms were isolated in 150 infectious episodes (66.1%), 21 (14%) of which were polymicrobial. Among those treated with ceftobiprole-M, microorganisms were isolated in 139 episodes (65.5%), of which 53 (38.1%) were polymicrobial, a statistically significant difference (*p* = 0.0001) (Table 3).

Microorganisms isolated in ceftaroline-F-treated patients were GPC in 139 (92.7%), thirty-nine (26%) of which were MRSA; methicillin-susceptible *Staphylococcus aureus* (MSSA) in thirty-one (20.7%); GNB in nine (6%), of which eight (5.3%) were multi-susceptible Enterobacteriaceae (five *Escherichia coli*, two *Klebsiella pneumoniae*, and one *Providencia stuarti*); and nonfermenting GNB (*Hemophilus influenzae*) in one (Supplementary Table S2). Microorganisms isolated in ceftobiprole-M-treated patients were GPC in ninety (64.7%), of which twenty-five (18%) were MRSA and twenty-one (15.1%) were MSSA; methicillin-resistant CoNS in forty (26.7%); and GNB in forty-eight (19.7%), of which twekve (8.6%) were multi-susceptible Enterobacteriaceae (four *Escherichia coli*, five *Klebsiella pneumoniae*, one *Proteus vulgaris*, one *Proteus mirabilis*, and one *Klebsiella oxytoca*), *Pseudomonas aeruginosa* in thirty-two (23%), and other nonfermenting GNB in four (2.9%) (two *Morganella morganii*, one *Moraxella catarrhalis*, and one *Haemophilus influenzae*).

All GPC isolates in the ceftaroline-F group (*n* = 113) were susceptible to vancomycin vs. 96.4% of GPC isolates in the ceftobiprole-M group (*n* = 53) (*p* = 0.106), with one *Staphylococcus hominis* and one MRSA being vancomycin resistant.

All tested microorganisms in the ceftaroline-F group (forty-three microorganisms: thirteen MRSA, 12 MSSA, eleven *Staphylococcus epidermidis*, three *Staphylococcus hominis*, two *Staphylococcus lungdunensis*, one *Staphylococcus capitis*, and one *Staphylococcus xylosus*) and in the ceftobiprole-M group (twenty-one microorganisms: three MSSA, three MRSA, four *Pseudomonas aeruginosa*, two *Staphylococcus epidermidis*, one *Staphylococcus haemolyticus*, one *Proteus mirabilis*, one *Klebsiella pneumoniae*, one *Escherichia coli*, one *Streptococcus anginosus*, one *Enterococcus faecalis*, one *Morganella morganii*, one *Proteus vulgaris*, and one *Klebsiella oxytoca*) were susceptible to both antibiotics.

### 2.3. Health Outcomes

Patients treated with ceftaroline-F had a longer hospital stay (36 days [IQR 19–60] vs. 19.5 days (IQR 12–30.75)). Total crude mortality and infection-related mortality rates were higher in the ceftaroline-F group (33.9 vs. 21.2%, *p* = 0.003, 18.9 vs. 11.8%, *p* = 0.039, respectively), although there was no difference in the mortality at 14 (13.2 vs. 8.0%, *p* = 0.078) or 28 days (4.8 vs. 3.3%, *p* = 0.415). A lower percentage of hospital readmissions for the same motive was observed in ceftaroline-Fvs. ceftobiprole-M-treated patients (1.3 vs. 4.7%, *p* 0.003). Relapses were recorded in 0% of ceftaroline-F-treated patients vs. 1.4% of ceftobiprole-M-treated patients (*p* = 0.072) (Table 4). 

**Figure 1:**
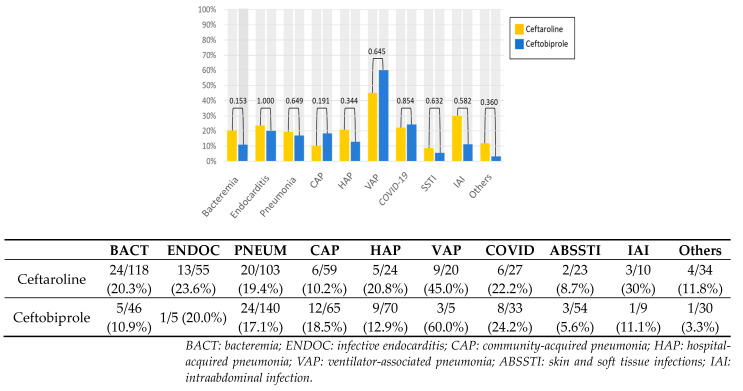
Infection-related mortality by infection pathway.

**Figure 2:**
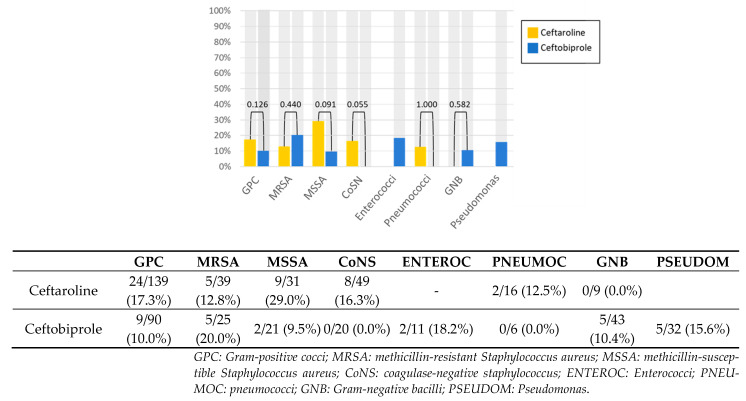
Mortality by microbial isolate.

The infection-related mortality rate did not differ between the antibiotics by type of infection (Figure 1) or microbiological isolate (Figure 2). 

#### Infective Endocarditis 

Among the sixty patients with IE, fifty-five (91.6%) received ceftaroline-F and only five (8.3%) received ceftobiprole-M. Defined IE was present in 37 (67.3%) of the patients treated with ceftaroline-F. The IE type most frequently treated with ceftaroline-F was native valve-related (45.5%), followed by early prosthetic (18.2%), late prosthetic (12.7%), and device-related (10.9%) IE; surgery was performed in 21 ceftaroline-F-treated patients with IE (38.2%) and was prescribed but not performed in 11 (20%) due to their clinical status or technical infeasibility. The most frequently isolated microorganisms in these patients were SA (40%), with an MRSA rate of 27.3%, and CoNS (38.2%), with a *Staphylococcus epidermidis* rate of 29.1%. The global mortality rate was 40%, and the infection-related mortality rate was 23.6% (Table 5).

### 2.4. Adverse Effects

No adverse effects were observed in 96% of the patients treated with ceftaroline-F or 97.6% of those treated with ceftobiprole-M. In the former, mild effects were recorded in three patients (1.3%) and moderate effects in six (2.5%), while withdrawal of the antibiotic was mandated in five (2.1%). In the five patients treated with ceftobiprole-M, adverse effects were mild in three (1.4%), moderate in two (0.9%), and caused withdrawal of the antibiotic in two (0.9%) (Table 6).

### 2.5. Mortality Risk Factors by Antibiotic 

Infection-related mortality risk factors in the ceftaroline-F group were a history of cardiovascular disease (53.5 vs. 24.0%, *p* 0.018), bacteremia (54.5 vs. 17.2%, *p* 0.001), IE (29.5 vs. 3.4%, *p* 0.006), and infection by GPC (100 vs. 64.3%, *p* = 0.002). Infection-related mortality risk factors in the ceftobiprole-M group were advanced age (75.88 ± 13.64 years vs. 65.84 ± 10.50 years, *p* = 0.001), the presence of CAP (41.4 vs. 13.6%, *p* = 0.007) or NP (31.0 vs. 11.4%, *p* = 0.037), polymicrobial infection (50.0 vs. 8.3%, *p* = 0.004), infection due to GNB (35.7 vs. 0.0%, *p* = 0.004) or *Pseudomonas* (35.7 vs. 0.0%, *p* = 0.002), monotherapy (56.0 vs. 7.0%, *p* = 0.0001), and empirical administration (84.0 vs. 46.5%, *p* 0.002). In the multivariate analysis, no differences were found between ceftarolineand ceftobiprole-treated patients whose death was directly attributable to their infection (Table 7).

## 3. Discussion

The patients in our cohort were mostly males with a high comorbidity index, and all were aged > 60 years. The ceftaroline-treated patients were younger, their clinical status was worse, and they more frequently received targeted and combined second-line treatments or more. Both ceftaroline and ceftobiprole were most often combined with daptomycin. Previous studies have demonstrated the clinical benefit of combining these fifth-generation cephalosporins with daptomycin in bacteriemia due to MRSA [21,22]. Around one in four patients were hospitalized in medical departments, with 20% of ceftaroline-F-treated patients and 5% of ceftobiprole-M-treated patients being in the ICU. More than half of the infections were nosocomial or healthcare-related, and around one-third of them met the criteria for sepsis. Septic shock, reported by some authors to increase the mortality risk by up to 40% [23], was five-fold more prevalent among the ceftaroline-F-treated patients in our cohort. 

Ceftaroline-F was most frequently prescribed to treat bacteremia, IE, ABSSTIs, or pneumonia. The IMVAP healing rate was 80%, similar to previous reports [24]. In comparison to the rates obtained with ceftaroline-F treatment in the multicenter CAPTURE study of more than 500 patients with pneumonia, the cure rate was slightly higher in the present patients with CAP (89.2 vs. 79%), lower in those with chronic obstructive pulmonary disease (COPD) (15 vs. 40%) [24], and similar in those with NP (80%) and IMVAP (60%) [25]. In the patients with IE treated with ceftaroline-F, the related mortality was 23.6%, lower than in the CAPTURE study (34.3%) [26], despite having a similar percentage of patients with IE due to SA (40 vs. 35.8%, respectively), and in surgery-indicated patients who did not undergo surgery (20% vs. 19.5%). It has previously been observed [27] that the isolation of MSSA and the indication but non-performance of surgery are associated with a higher mortality in patients with IE.

Ceftobiprole-M was prescribed against pneumonia in more than half of the patients, ABSSTIs in around one-fifth, and bacteremia in around one-sixth. Among the cases of pneumonia treated with ceftobiprole-M, around half were CAP and half NP, with only five cases of IMVAP. This fifth-generation cephalosporin has demonstrated non-inferior clinical effectiveness and safety with respect to its comparators in various clinical trials for the treatment of ABSSTIs [28], complicated *S. aureus* bacteriemia (ERADICATE study) [29], CAP, and NP [16,17]. However, worse outcomes have been obtained in patients with IMVAP treated with ceftobiprole-M vs. ceftazidime plus linezolid [17], and ceftobiprole-M is, therefore, not indicated for its treatment [30]. Recently, a real-life study has reported on the use of ceftobiprole-M for severe infections in a French ICU, finding that the main indication for this antibiotic was pneumonia (51%) with associated bacteremia (72%); clinical cure was achieved in around 80% of patients, with an in-hospital mortality rate of 32% [31].

One in ten patients with pneumonia in our study, including both ceftobiprole-Mand ceftaroline-F-treated patients, were coinfected with COVID-19. COVID-19 coinfection and suprainfection have been associated with a worse clinical prognosis in patients with respiratory infection, increasing the mortality risk by up to three-fold [32]. 

The infection-related mortality rate at 14 and 18 days did not differ between the ceftarolineand ceftobiprole-treated patients, and no between-group differences in age, sex, previous disease, infection type, or isolated microorganism were observed among those whose death was attributable to the infection. Mortality risk factors were older age, Charlson index, ICU stay, and the presence of sepsis and septic shock in both antibiotic groups. This is consistent with published evidence that age, comorbidity, ICU admission (and duration), and septic shock are independent prognostic factors for mortality in patients with sepsis [21,33,34]. The mortality rate of patients with IE treated with ceftaroline-F was 23.6%, although SA was the causal agent in two-fifths of these patients, and surgery was prescribed but not feasible in one-fifth. A previous study of patients with IE due to SA in our setting described a higher mortality rate of 50% that was not influenced by methicillin resistance [35]. 

The readmission rate was significantly higher among patients treated with ceftobiprole-M but below 5% for both antibiotics (4.7 vs. 1.3%, *p* = 0.036). Seven of the ten ceftobiprole-M-treated patients readmitted had ABSSTIs, which have previously been associated with a recurrence rate of around 10% [36]. However, interpretation of the results should take account of disparities between the antibiotic cohorts (in previous diseases, pathways, and microorganisms) that may diminish the comparability of outcomes.

No adverse effects were observed in 96% of patients receiving ceftaroline-F or in 97.6% of those receiving ceftobiprole-M. These data are similar to the findings of clinical trials [8,17,37] and real-life studies [22,38,39], in which the most frequent effects in these studies were gastrointestinal, cutaneous, and hepatic profile disorders, with the less common observation of myoclonia with ceftobiprole-M [38] and cytopenia with ceftaroline-F [40]. Cytopenia was observed in three of our ceftaroline-F-treated patients. Currently, a retrospective, single-center study has just been published, conducted in an Italian hospital, which compared in real life the effectiveness of the use of ceftaroline-M vs. ceftobiprole in a reduced number of subjects (75 vs. 63 patients), with data similar to ours in terms of clinical efficacy and tolerability [41]. 

This study is limited by its retrospective design and associated selection bias, which may influence the results obtained and their interpretation. However, this is the first real-life comparative study of ceftaroline-F and ceftobiprole-M and has a wide patient sample from different centers, representing as far as possible the routine clinical utilization of these fifth-generation cephalosporins in Spain. 

## 4. Materials and Methods

### 4.1. Study Design 

A real-life, retrospective, multicenter, observational, descriptive study was conducted on the use of ceftaroline-F and ceftobiprole-M in hospitalized patients or outpatient parenteral antimicrobial therapy (OPAT) with nosocomial/nosohusial or community-acquired infections. Patients were included between the date that these drugs were incorporated in the clinical protocol of the participating hospital and 31 July 2022. The study was approved by the provincial ethics committee of Granada (Ref. 0095-N-22) and deemed exempt from the requirement for informed patient consent. 

### 4.2. Treatment Description 

This descriptive study did not involve any pharmacological intervention, and all treatments were prescribed by the attending physicians according to their habitual criteria. Inclusion criteria were age ≥ 18 years; at least 48 h of antibiotic treatment with ceftaroline-F or ceftobiprole-M (≥6 vials in patients with normal renal function and creatinine clearance-adjusted dosage in patients with kidney failure) between the date of drug approval at the hospital and 31 July 2022; and the completion of a 28-day follow-up, except for patients with IE or osteomyelitis (6-month follow-up). Exclusion criteria were pregnancy and allergy to beta-lactams or some formulation excipient.

### 4.3. Variables

Data were gathered on sex, age, hospital stay, prescribing hospital department, patient comorbidities, infection acquisition pathway, and the presence of sepsis or septic shock. The age-corrected Charlson index was calculated for each patient [42].

Information was also collected on the number of active infectious foci, the presence of bacteremia (primary/catheter-related) and IE (by modified Duke’s diagnostic criteria [43] (defined/possible/rejected–suspicion), the type of IE (native valve/early prosthetic/late prosthetic/pacemaker or device/transcatheter aortic valve implantation, the performance of surgery (not indicated/indicated but not undergone/device extraction)), and the presence of respiratory infection (CAP/NP/invasive mechanical ventilation-associated pneumonia (IMVAP)), ABSSTI, urinary tract infection, central nervous system infection, spondylodiscitis, osteoarticular infection, intraabdominal infection, fever with no focus, and other infectious foci. For respiratory infections, data were recorded on coinfection by coronavirus SARS-2 2019 and suspected or confirmed fungal coinfection. 

Treatment details were collected on the prescribed antibiotic (ceftaroline-F or ceftobiprole-M); the mode of administration, monotherapy or a combination with other antibiotics against the same infection, empirical or targeted, first-line or rescue treatment (in the latter case, specifying previous antibiotic therapy for the same infection and its duration); reason for antibiotic rescue (previous antibiotic treatment failure/receipt of indicative microbiological results/emergence of toxicity or adverse effects with previous treatments) and appropriate empirical antibiotic therapy (i.e., effective against isolated microorganisms or good clinical outcome if none are isolated); and the total days of antibiotic administration, total administered dose, and the presence, type, and severity of adverse effects.

Microbiology data were collected on the monoor poly-microbial nature of the infection; the microorganism(s) identified; and the antibiogram, in accordance with EUCAST criteria [44]. MIC cutoffs (mg/L) by genus were *Staphylococcus* (two vancomycin (SA), five vancomycin (CoNS), two oxacillin (SA), 0.25 oxacillin (CoNS)); *Enterococcus* (four vancomycin); *Streptococcus pneumoniae* (1 cefepime; 0.5 ceftobiprole; two vancomycin; two meropenem); *Enterobacteriaceae* (1 cefepime; 0.25 ceftobiprole; two meropenem); and *Pseudomonas aeruginosa* (0.001 cefepime; ceftobiprole: insufficient evidence; two meropenem). The studied microorganisms were isolates obtained in conventional culture media, including hemocultures and cultures of urine, cerebrospinal fluid, bone, and abscesses from patients during an infectious episode treated with either antibiotic. 

The following health outcomes were recorded: clinical cure, total crude mortality at 14 and 28 days (6 months for patients with IE or osteomyelitis), infection-related mortality, readmission for the same motive, and infection recurrence.

### 4.4. Definitions

Nosocomial/Nosohusial infection: infection in patients hospitalized for >72 h (nosocomial) or healthcare-related infection (day hospital, nursing home, day center for the elderly). Septic shock: refractory hypotension accompanied by hypoperfusion and organ dysfunction despite adequate fluid resuscitation [45]. Immunosuppression: congenital or acquired immunodeficiency (including oncohematological patients) or secondary to immunosuppressive treatment [46]. Recurrence/relapse: the second microbiologically confirmed episode of the infection within one month of discharge [47]. Severity of adverse effects: mild = no requirement for antidote or treatment; moderate = requirement for treatment modification (e.g., dose adjustment, combination with other drugs) but not an interruption, with possible need for a longer hospital stay or specific treatment prescription; severe = life threatening, mandating an interruption of drug administration and the prescription of a specific treatment; or lethal = directly or indirectly contributing to death. 

Data were gathered from the different electronic records systems of participating hospitals by obtaining the records of patients receiving the drugs from the hospital Pharmacy Departments. Data were anonymized before being entered into the SPSS 20.0 database in accordance with the Helsinki Declaration and Spanish data protection legislation (Law 3/2018 5 December).

### 4.5. Sample Size

This descriptive comparative study included all patients meeting study eligibility criteria treated with ceftaroline-F or ceftobiprole-M at participating centers during the study period.

### 4.6. Statistical Analysis 

In a descriptive analysis, absolute and relative frequencies (%) with a 95% confidence interval (CI) were calculated for qualitative variables, means with standard deviation (SD) for quantitative variables with a normal distribution (Kolmogorov–Smirnov test), and medians with an interquartile range (IQR) for those with a non-normal distribution. In bivariate analyses, the chi-square test was used to compare qualitative variables, Student’s t-test was used for quantitative variables with normal distributions, and the Mann–Whitney U test was used for quantitative variables with non-normal distributions.

A logistic regression multivariate analysis was performed to examine between-group differences in epidemiologic variables, medical history of interest, drug dose, and drug administration (alone/in combination) and its prescription as empirical, targeted, or rescue therapy (with reason for rescue) by entering all variables that were statistically significant in bivariate analysis. Another regression analysis was carried out to evaluate mortality-related factors in the two antibiotic groups. 

## 5. Conclusions

The fifth-generation cephalosporins, ceftaroline-F and ceftobiprole-M, are safe and effective in real life, with no difference between them in health outcomes.

## Figures and Tables

**Table 1:** Epidemiological characteristics, comorbidities, and antibiotic therapy.

	Ceftaroline (Cohort *N* = 227)	Ceftobiprole (Cohort *N* = 212)	*p* *	OR (95%CI)
Age, mean (years) (± SD)	63.02 (±13.45)	66.40 (±15.44)	0.015	1.1 (1.001–1.05)
Charlson index, mean (IQR)	4 (2–6)	4 (2–6)	0.290	
Men, *n* (%)	140 (61.7)	126 (59.4)	0.631	
Sepsis or septic shock, *n* (%)	86 (37.9)	62 (29.2)	0.056	
- Sepsis	38 (16.7)	53 (25.0)	0.033	6.7 (1.96–22.9)
- Septic shock	48 (21.1)	8 (4.2)	0.0001	0.27 (0.09–0.81)
Acquisition of infection, *n* (%)				
- Community-acquired infection	109 (48.0)	91 (42.9)	0.284	
- Nosocomial/Nosohusial infection	118 (52.0)	121 (57.1)		
Inpatient department, *n* (%)	191 (84.1)	169 (79.7)	0.228	
Medical departments, *n* (%)	119 (62.3)	134 (78.1)	0.234	
Surgical department, *n* (%)	36 (18.8)	35 (20.7)	0.853	
OPAT, *n* (%)	0 (0.0)	8 (3.8)	0.003	0 (0–0)
Cardiovascular risk factors, *n* (%)	168 (74.0)	104 (49.1)	0.0001	0.66 (0.19–2.3)
- Arterial hypertension	123 (54.2)	91 (42.9)	0.018	1.4 (0.45–3.9)
- Diabetes mellitus	82 (36.1)	55 (25.9)	0.021	1.04 (0.5–2.2)
- Dyslipidemia	80 (35.2)	31 (14.6)	0.0001	0.38 (0.16–0.87)
- Hyperuricemia	21 (9.3)	1 (0.5)	0.0001	0 (0–0)
- Obesity	30 (13.2)	9 (4.2)	0.001	0.41 (0.12–1.42)
- Obstructive sleep apnea syndrome	18 (7.9)	6 (2.8)	0.019	0.52 (0.11–2.4)
Cardiovascular diseases, *n* (%)	90 (39.6)	68 (32.1)	0.099	
- Heart failure	33 (14.5)	21 (9.9)	0.140	
- Ischemic heart disease	34 (15.0)	33 (15.6)	0.864	
- Moderate-severe valve disease	43 (18.9)	9 (4.2)	0.0001	0.26 (0.08–0.82)
- Atrial fibrillation/flutter	37 (16.3)	18 (8.5)	0.014	1.2 (0.45–3.35)
Respiratory diseases, *n* (%)	56 (24.7)	45 (21.2)	0.392	
- COPD	32 (14.1)	36 (17.0)	0.404	
- Bronchiectasis	10 (4.4)	11 (5.2)	0.701	
Cirrhosis—chronic liver disease, *n* (%)	21 (9.3)	23 (10.8)	0.577	
Chronic kidney disease, *n* (%)	35 (15.4)	27 (12.7)	0.420	
- Dialysis	7 (3.1)	6 (2.8)	0.876	
Active solid malignancy, *n* (%)	10 (4.4)	15 (7.1)	0.152	
- Active treatment	6 (2.6)	15 (7.1)	0.030	2.9 (0.9–8.8)
- Progression/Palliative care	4 (1.8)	0 (0.0)	0.090	
Active hematologic disease, *n* (%)	15 (6.6)	23 (10.8)	0.114	
Neurological disease, *n* (%)	30 (13.2)	37 (17.5)	0.217	
- Dementia/Cognitive impairment	6 (2.6)	11 (5.2)	0.167	
- Stroke	19 (8.4)	15 (7.1)	0.612	
- Parkinson’s disease	3 (1.3)	3 (1.4)	1.000	
Immunocompromised, *n* (%)	60 (26.4)	61 (28.8)	0.583	
- Immunosuppressive drug therapy	45 (19.8)	35 (16.6)	0.381	
Solid organ transplantation, *n* (%)	23 (10.1)	5 (2.4)	0.001	
HIV infection, *n* (%)	4 (1.8)	7 (3.3)	0.302	
Antibiotic therapy				
Total dose of antibiotic (mg), median (IQR)	11,700 (6600–19,800)	11,000 (6250–15,000)	0.015	
Duration of antibiotic therapy (days), median (IQR)	8 (5–14)	7 (5–10)	0.023	
Treatment regimen, *n* (%)				
- Monotherapy	25 (11.0)	115 (54.2)	0.0001	0.13 (0.06–0.28)
- Antibiotic combination	202 (89.0)	97 (45.8)		
Susceptibility-guided treatment, *n* (%)	131 (57.7)	63 (29.7)	0.0001	0.35 (0.18–0.69)
Empirical treatment, *n* (%)	96 (42.3)	149 (70.3)	0.104	
Antibiotic prescription, *n* (%)				
- As a first-line treatment	40 (17.6)	75 (35.4)	0.0001	0.35 (0.18–0.69)
- As a second-line or more treatment	187 (82.4)	137 (64.6)		
Duration of previous antibiotic treatment (days), median (IQR)	6 (3–10)	3 (2–6.50)	0.0001	1 (1–1)
Reason for switching, *n* (%)				
- Failure of previous antibiotic	152 (81.3)	92 (67.2)	0.004	1.3 (0.32–5.4)
- Guided by microbiological results	22 (11.8)	39 (28.5)	0.0001	6.3 (1.3–30.5)
- Toxicity/Adverse effects of previous antibiotic treatment	13 (7.0)	6 (4.4)	0.330	

OPAT: outpatient parenteral antibiotic treatment; COPD: chronic obstructive pulmonary disease, HIV: human immunodeficiency virus, SD: standard deviation, IQR: interquartile range, *p* * < 0.05 = significant.

**Table 2:** Infection pathways.

	Ceftaroline (Cohort *N* = 227)	Ceftobiprole (Cohort *N* = 212)	*p* *
Number of infective foci, median (IQR)	1 (1–1)	1 (1–1)	0.007
Bloodstream infections, *n* (%)	118 (51.9)	46 (21.7)	0.0001
- Primary bacteremia	31 (13.7)	34 (16)	0.867
- Catheter-associated bacteremia	27 (11.9)	7 (3.3)	0.0001
Infective endocarditis, *n* (%)	55 (24.2)	5 (2.4)	0.0001
Respiratory tract infections, *n* (%)	103 (45.4)	140 (66)	0.006
- Community-acquired pneumonia	59 (25.9)	65 (30.7)	0.780
- Nosocomial pneumonia	24 (10.6)	70 (33)	0.0001
- Ventilator-associated pneumonia	20 (8.8)	5 (2.4)	0.001
- Coinfection with SARS-CoV-2	27 (11.9)	34 (16)	0.545
Soft tissue and skin infection, *n* (%)	23 (10.1)	54 (25.4)	0.0001
- Cellulitis	4 (1.8)	10 (4.7)	
- Myositis	6 (2.6)	5 (2.4)	
- Soft tissue abscess	4 (1.8)	7 (3.3)	
- Diabetic foot infection	1 (0.4)	20 (9.4)	
- Infected pressure ulcer	2 (0.7)	7 (3.3)	
- Surgical wound infection	6 (2.6)	5 (2.4)	
Urinary tract infection, *n* (%)	6 (2.6)	10 (4.7)	0.277
- Non-complicated UTI	3 (1.3)	3 (1.4)	
- Complicated UTI (pyelonephritis)	3 (1.3)	5 (2.4)	
- Renal abscess	0 (0.0)	2 (0.9)	
Central nervous system infection, *n* (%)	9 (3.9)	8 (3.8)	0.719
- Meningitis	3 (1.3)	1 (0.5)	
- Cerebral abscess	0 (0.0)	2 (0.9)	
- Ventriculoperitoneal shunt infection	2 (0.7)	3 (1.4)	
- Epidural abscess	4 (1.8)	2 (0.9)	
Intraabdominal infection, *n* (%)	10 (4.4)	9 (4.2)	0.724
Bone and joint infection, *n* (%)	13 (5.7)	11 (5.2)	0.580
- Osteomyelitis	6 (2.6)	5 (2.4)	
- Septic arthritis	7 (3.1)	1 (0.5)	
- Prosthetic joint infection	0 (0.0)	5 (2.4)	
Spondylodiscitis, *n* (%)	9 (3.9)	4 (1.9)	0.133
Fever without focus, *n* (%)	8 (3.5)	0 (0.0)	0.003
Other type of infection, *n* (%)	6 (2.6)	4 (1.9)	0.468

UTI: urinary tract infection, *p* * < 0.05 = significant.

**Table 3:** Microbiological isolates.

Positive Microbial Samples	Ceftaroline (Cohort *N* = 150)	Ceftobiprole (Cohort *N* = 139)	p *
Microbial profile of isolates, *n* (%)			
- Monomicrobial infection	129 (86.0)	86 (61.9)	0.0001
- Polymicrobial infection	21 (14.0)	53 (38.1)	
Gram-positive cocci, *n* (%)	139 (92.7)	90 (64.7)	0.0001
MR *Staphylococcus aureus*	39 (26.0)	25 (18.0)	0.101
MS *Staphylococcus aureus*	31 (20.7)	21 (15.1)	0.219
MR CoNS	40 (26.7)	14 (10.1)	0.0001
- *Staphylococcus epidermidi*s	31 (20.7)	12 (8.6)	
- *Staphylococcus hominis*	3 (2.0)	1 (0.7)	
- *Staphylococcus haemolyticus*	4 (1.3)	1 (0.7)	
- *Staphylococcus capitis*	1 (0.7)	0 (0.0)	
- *Staphylococcus xylosus*	1 (0.7)	0 (0.0)	
MS CoNS	9 (6.0)	6 (4.3)	0.519
- *Staphylococcus epidermidis*	3 (2.0)	3 (2.2)	
- *Staphylococcus hominis*	2 (1.3)	1 (0.7)	
- *Staphylococcus haemolyticus*	0 (0.0)	1 (0.7)	
- *Staphylococcus lugdunensis*	3 (2.0)	0 (0.0)	
- *Staphylococcus schleiferi*	0 (0.0)	1 (0.7)	
- *Staphylococcus saccharomyces*	1 (0.7)	1 (0.7)	
*Enterococcus faecium*	0 (0.0)	1 (0.7)	0.481
*Enterococcus faecalis*	0 (0.0)	10 (7.2)	0.001
*Streptococcus pneumoniae*	16 (10.7)	6 (4.3)	0.042
Other *Streptococcus species*	4 (2.7)	4 (3.6)	0.742
- *Streptococcus pyogenes*	1 (0.7)	0 (0.0)	
- *Streptococcus anginosus*	1 (0.7)	3 (2.2)	
- *Streptococcus agalactiae*	1 (0.7)	0 (0.0)	
- *Streptococcus peroris*	0 (0.0)	1 (0.7)	
- *Streptococcus mitis*	1 (0.7)	0 (0.0)	
*Rothia* spp.	0 (0.0)	2 (1.4)	0.230
Gram-positive bacilli, *n* (%)	2 (1.3)	1 (0.7)	1.000
- *Corynebacterium coylea*e	1 (0.7)	0 (0.0)	
- *Lactobacillus rhamnosus*	1 (0.7)	0 (0.0)	
- *Cutibacterium acnes*	0 (0.0)	1 (0.7)	
Gram-negative bacilli, *n* (%)	9 (6.0)	48 (19.7)	0.0001
Enterobacterales	8 (5.3)	12 (8.6)	0.188
- *Escherichia coli*	5 (3.3)	4 (2.9)	
- *Klebsiella pneumoniae*	2 (1.3)	5 (3.6)	
- *Proteus vulgaris*	0 (0.0)	1 (0.7)	
- *Proteus mirabilis*	0 (0.0)	1 (0.7)	
- *Providencia stuartii*	1 (0.7)	0 (0.0)	
- *Klebsiella oxytoca*	0 (0.0)	1 (0.7)	
*Pseudomonas aeruginosa*	0 (0.0)	32 (23.0)	0.0001
Other Gram-negative bacilli	1 (0.7)	4 (2.9)	0.199
- *Morganella morganii*	0 (0.0)	2 (1.3)	
- *Moraxella catarrhalis*	0 (0.0)	1 (0.7)	
- *Haemophilus influenzae*	1 (0.7)	1 (0.7)	

MR: methicillin resistant, MS: methicillin susceptible, CoNS: coagulase-negative staphylococci, *p* * < 0.05 = significant.

**Table 4:** Outcomes.

	Ceftaroline (*N* = 227)	Ceftobiprole (*N* = 212)	*p* *
Length of hospital stay (days), median (IQR)	36 (19–60)	19.5 (12–30.7)	0.0001
Readmission and recurrence, *n* (%)			
- Readmission	3 (1.3)	10 (4.7)	0.036
- Recurrence of infection (in the first month)	0 (0.0)	3 (1.4)	0.072
Mortality, *n* (%)			
- Total mortality	77 (33.9)	45 (21.2)	0.003
- Infection-related mortality	43 (18.9)	25 (11.8)	0.039
Infection-related mortality, *n* (%)			
- 14-day mortality	30 (13.2)	17 (8.0)	0.078
- 28-day mortality	11 (4.8)	7 (3.3)	0.415
- 6-month mortality	2 (0.9)	1 (0.5)	1.000

IQR: interquartile range, *p* * < 0.05 = significant.

**Table 5:** Infective endocarditis: characteristics, antibiotic therapy, and outcomes.

	Ceftaroline (Cohort *N* = 55)	Ceftobiprole (Cohort *N* = 5)
Age, mean (years) (± SD)	69.33 (±11.62)	67.20 (±2.39)
Charlson index, mean (IQR)	5 (3–6)	4 (4–4.50)
Number of infective foci, median (IQR)	1 (1–1)	2 (1–2)
Definition (modified Duke criteria), *n*(%)		
- Defined	37 (67.3)	1 (20)
- Possible	8 (14.5)	1 (20)
- Suspected	10 (18.2)	3 (60)
Location of infective endocarditis, *n* (%)		
- Native valve	25 (45.5)	2 (40)
- Early prosthetic valve	10 (18.2)	0 (0.0)
- Late prosthetic valve	7 (12.7)	1 (20)
- Pacemaker device	6 (10.9)	0 (0.0)
- Prosthetic valve post-TAVI	1 (1.8)	0 (0.0)
Cardiac surgery, *n* (%)		
- Not indicated	9 (16.4)	0 (0.0)
- Surgery undergone	21 (38.2)	1 (20)
- Indicated but not undergone	11 (20.0)	1 (20)
- Device extraction	5 (9.0)	0 (0.0)
Sepsis or septic shock, *n* (%)	21 (38.2)	3 (60)
Microbiological isolation, *n* (%)	46 (83.6)	5 (100.0)
Staphylococcus aureus	22 (40.0)	1 (20.0)
- MSSA	7 (12.7)	1 (20.0)
- MRSA	15 (27.3)	0 (0.0)
CoNS	22 (40.0)	1 (20.0)
Enterococcus faecalis	0 (0.0)	3 (60.0)
Corynebacterium coyleae	1 (1.8)	0 (0.0)
Streptococcus pneumoniae	1 (1.8)	0 (0.0)
Antibiotic combination, *n* (%)	55 (100)	5 (100)
- Combined with cloxacillin	6 (10.9)	1 (20.0)
- Combined with ampicillin	3 (5.5)	2 (40.0)
- Combined with daptomycin	42 (76.4)	2 (40.0)
Antibiotic prescription, *n* (%)		
- As a first-line treatment	7 (12.7)	1 (20)
- As a second-line or more treatment	48 (87.3)	4 (80)
Reason for switching, *n* (%)		
- Failure of previous antibiotic treatment	36 (75.0)	2 (50.0)
- Guided by microbiological results	8 (16.7)	2 (50.0)
- Toxicity/adverse effects of previous antibiotic treatment	4 (8.3)	0 (0.0)
Total dose of antibiotic (mg), median (IQR)	16,200 (9200–39,600)	54,400 (7000–60,000)
Duration of antibiotic therapy (days), median (IQR)	13 (6–23)	28 (4.50–40)
Length of hospital stay (days), median (IQR)	38 (20–64)	46 (35–100)
Total mortality, *n* (%)	22 (40)	1 (20)
Related mortality, *n* (%)	13 (23.6)	1 (20)
- 14-day mortality	8 (14.5)	1 (20)
- 28-day mortality	3 (5.5)	0 (0.0)
- 6-month mortality	2 (3.6)	0 (0.0)
Readmission, *n* (%)	1 (1.8)	0 (0.0)

TAVI: transcatheter aortic valve implantation, MSSA: methicillin-susceptible *Staphylococcus aureus*, MRSA: methicillin-resistant *Staphylococcus aureus*, CoNS: coagulase-negative staphylococci, IQR: interquartile range, SD: standard deviation.

**Table 6:** Adverse drug effects.

	Ceftaroline (*N* = 227)	Ceftobiprole (*N* = 212)	*p* *
Total adverse effects, *n* (%)	9 (4.0)	5 (2.4)	0.338
Severity of adverse effects, *n* (%)			
- Mild	3 (1.3)	3 (1.4)	
- Moderate	6 (2.6)	2 (0.9)	
- Severe	0 (0.0)	0 (0.0)	
- Lethal	0 (0.0)	0 (0.0)	
Antibiotic withdrawal for adverse effects, *n* (%)	5 (2.2)	2 (0.9)	1.000
Adverse effects by symptoms, *n* (%)			
- Urticaria-like cutaneous rash	2 (0.9)	1 (0.5)	1.000
- Elevated liver enzymes (hepatocellular pattern)	2 (0.9)	3 (1.4)	0.266
- Elevated liver enzymes (cholestatic pattern)	1 (0.4)	1 (0.5)	1.000
- Gastrointestinal symptoms	1 (0.4)	1 (0.5)	1.000
- Thrombocytopenia	1 (0.4)	0 (0.0)	1.000
- Neutropenia	2 (0.9)	0 (0.0)	0.505
- Fever and eosinophilia	2 (0.9)	0 (0.0)	0.505

*p* * < 0.05 = significant.

**Table 7:** Bivariate and multivariate analysis of infection-related mortality.

	Deaths in Ceftaroline Group (*N* = 43)	Deaths in Ceftobiprole Group (*N* = 25)	*p* *	OR (95%CI)
Age, mean (years) (± SD)	65.84 (±10.50)	75.88 (±13.64)	0.001	0.9 (0.81–1.2)
Charlson index, median (IQR)	5 (4–7)	4 (4–6.50)	0.330	
Men, *n* (%)	29 (67.4)	17 (68.0)	0.962	
Sepsis	25 (58.1)	14 (56.0)	0.863	
Septic shock	19 (44.2)	7 (28.0)	0.185	
Infection acquisition, *n* (%)				
Community acquired	15 (34.9)	11 (44.0)	0.456	
Nosocomial/Nosohusial	28 (65.1)	14 (56.0)		
Inpatient department, *n* (%)				
Medical department	40 (93.0)	22 (88.0)	0.481	
Surgical department	3 (7.0)	2 (8.0)	0.876	
OPAT, *n* (%)	0 (0.0)	1 (4.0)	0.186	
Comorbidities, *n* (%)				
Cardiovascular risk factors	36 (83.7)	19 (76.0)	0.435	
Obesity	8 (18.6)	0 (0.0)	0.022	0 (0–0)
Cardiovascular diseases	23 (53.5)	6 (24.0)	0.018	3.1 (0.2–69)
Ischemic heart disease	11 (25.6)	1 (4.0)	0.024	0.15 (0.001–27)
Moderate-severe valve disease	12 (27.9)	0 (0.0)	0.004	0 (0–0)
Respiratory diseases	13 (30.2)	6 (24.0)	0.581	
Cirrhosis—chronic liver disease	3 (7.0)	2 (8.0)	1.000	
Chronic kidney disease	10 (23.3)	3 (12.0)	0.255	
Active solid malignancy	4 (9.3)	2 (8.0)	1.000	
Active hematologic disease	3 (7.0)	3 (12.0)	0.481	
Neurological disease	7 (16.3)	7 (28.0)	0.249	
Immunocompromised patients	16 (37.2)	10 (40.0)	0.819	
Infection pathways, *n* (%)				
Bloodstream infections	24 (54.5)	5 (17.2)	0.001	0.09 (0.004–2.7)
Infective endocarditis	13 (29.5)	1 (3.4)	0.006	8.6 (0.35–210)
Community-acquired pneumonia	6 (13.6)	12 (41.4)	0.007	0 (0–0)
Nosocomial pneumonia	5 (11.4)	9 (31.0)	0.037	
Ventilator-associated pneumonia	9 (20.5)	3 (10.3)	0.254	
Coinfection with SARS-CoV-2	6 (13.6)	8 (27.6)	0.139	
Fungal coinfection	5 (11.4)	0 (0.0)	0.150	
Soft tissue and skin infection	2 (4.5)	3 (10.3)	0.380	
Intraabdominal infection	3 (6.8)	1 (3.4)	0.925	
Central nervous system infection	1 (2.3)	0 (0.0)	1.000	
Bone and joint infection	1 (2.3)	1 (3.4)	1.000	
Spondylodiscitis	3 (6.8)	0 (0.0)	0.272	
Microbiological isolates, *n* (%)				
General microbial profile				
- No isolation	20 (45.5)	15 (51.7)	0.600	
- Positive microbial samples	24 (54.5)	14 (48.3)		
Microbial profile of isolates				
- Monomicrobial infection	22 (91.7)	7 (50.0)	0.004	2.6 (0.01–644)
- Polymicrobial infection	2 (8.3)	7 (50.0)		
Gram-positive cocci				
- MRSA	5 (20.8)	5 (35.7)	0.315	
- MSSA	9 (37.5)	2 (14.3)	0.128	
- MR CoNS	6 (25.0)	0 (0.0)	0.067	
- MS CoNS	2 (8.3)	0 (0.0)	0.522	
- *Enterococcus* spp.	0 (0.0)	2 (14.3)	0.129	
- *Streptococcus pneumoniae*	2 (8.3)	(0.0)	0.522	
Gram-negative bacilli	0 (0.0)	5 (35.7)	0.004	
Pseudomonas aeruginosa	0 (0.0)	5 (35.7)	0.002	
Antibiotic therapy				
Total dose (mg), median (IQR)	9600 (5400–14,400)	9000 (5000–12,500)	0.545	
Duration of antibiotic therapy (days), median (IQR)	7 (3–12)	6 (3–8.50)	0.527	
Antibiotic prescription, *n* (%)				
- As a first-line treatment	4 (9.3)	6 (24.0)	0.099	2.6 (0.02–391)
- As a second-line or more treatment	39 (90.7)	19 (76.0)		
Treatment regimen, *n* (%)				
- Monotherapy	3 (7.0)	17 (56.0)	0.0001	0 (0–0)
- Antibiotic combination	40 (93.0)	12 (44.0)		
Empirical treatment, *n* (%)	20 (46.5)	21 (84.0)	0.002	0.3 (0.01–6.7)

MRSA: methicillin-resistant *Staphylococcus aureus*, MSSA: methicillin-susceptible *S. aureus*, MR CoNS: methicillin-resistant coagulase-negative staphylococci, MS CoNS: methicillin-susceptible coagulase-negative staphylococci, OPAT: outpatient parenteral antibiotic therapy, SD: standard deviation, IQR: interquartile range, *p* * < 0.05 = significant.

## Data Availability

The authors confirm the accuracy and availability of the data used in this study.

## Supplementary Materials

The following supporting information can be downloaded at https://www.mdpi.com/article/10.3390/antibiotics12121692/s1, Table S1: Combined antibiotic therapy with ceftaroline or ceftobiprole; Table S2: Previous antibiotic therapy before prescription of ceftaroline or ceftobiprole.
