# Supplementary material for: Ceft-to-Ceft Study: Real-Life Experience with Ceftaroline and Ceftobiprole in Treatment of the Principal Infectious Syndromes in a Spanish Multicenter Hospital Cohort
Source: Antibiotics (Basel). 2023 Dec 2;12(12):1692. doi: 10.3390/antibiotics12121692 (PMC10740782; doi:10.3390/antibiotics12121692)
Supplement: Supplementary file 1 [file antibiotics-12-01692-s001.zip › antibiotics-2699663-supplementary.pdf]

Supplementary Table S1. Combined antibiotic therapy with Ceftaroline or Ceftobiprole

Combined antibiotic therapy	Ceftaroline (Cohort *N*=202)	Ceftobiprole (Cohort *N*=97)
Amikacin	3 (1.5%)	6 (6.2%)
Ampicillin	5 (2.5%)	8 (8.2%)
Azithromycin	15 (7.4%)	8 (8.2%)
Aztreonam	1 (0.5%)	0 (0.0%)
Cefepime	1 (0.5%)	0 (0.0%)
Ceftazidime-avibactam	13 (6.4%)	3 (3.1%)
Ceftolozane-tazobactam	4 (2.0%)	0 (0.0%)
Ceftriaxone	2 (1.0%)	1 (1.0%)
Ciprofloxacin	23 (11.4%)	3 (3.1%)
Clindamycin	2 (1.0%)	2 (2.1%)
Cloxacillin	14 (6.9%)	3 (3.1%)
Colistin	6 (3.0%)	0 (0.0%)
Cotrimoxazole	5 (2.5%)	6 (6.2%)
Dalbavancin	0 (0.0%)	1 (1.0%)
Daptomycin	80 (39.6%)	21 (21.6%)
Doxycycline	0 (0.0%)	2 (2.1%)
Fosfomycin	3 (1.5%)	1 (1.0%)
Levofloxacin	8 (4.0%)	7 (7.2%)
Linezolid	4 (2.0%)	7 (7.2%)
Meropenem	46 (22.8%)	6 (6.2%)
Metronidazole	1 (0.5%)	9 (9.3%)
Piperacillin-tazobactam	17 (8.4%)	1 (1.0%)
Rifampin	8 (4.0%)	0 (0.0%)
Tigecycline	2 (1.1%)	4 (4.1%)
Vancomycin	0 (0.0%)	4 (4.1%)

Supplementary Table S2. Previous antibiotic therapy before prescription of Ceftaroline or Ceftobiprole

Previous antibiotic therapy	Ceftaroline as second-line or more treatment (Cohort *N*=187)	Ceftobiprole as second-line or more treatment (Cohort *N*=137)
Amikacin	3 (1.6%)	3 (2.2%)
Amoxicillin-clavulanic acid	5 (2.7%)	1 (0.7%)
Ampicillin	3 (1.6%)	5 (3.6%)
Azithromycin	10 (5.3%)	5 (3.6%)
Cefazolin	1 (0.5%)	1 (0.7%)
Cefotaxime	2 (1.1%)	0 (0.0%)
Ceftazidime-avibactam	9 (4.8%)	1 (0.7%)
Ceftobiprole	1 (0.5%)	0 (0.0%)
Ceftolozane-tazobactam	2 (1.1%)	0 (0.0%)
Ceftriaxone	22 (11.8%)	14 (10.2%)
Ciprofloxacin	11 (5.9%)	2 (1.5%)
Clindamycin	2 (1.1%)	0 (0.0%)
Cloxacillin	22 (11.8%)	3 (2.2%)
Colistin	4 (2.1%)	0 (0.0%)
Cotrimoxazole	4 (2.1%)	4 (2.9%)
Daptomycin	62 (33.2%)	17 (12.4%)
Dalbavancin	0 (0.0%)	1 (0.7%)
Doxycycline	0 (0.0%)	1 (0.7%)
Ertapenem	1 (0.5%)	0 (0.0%)
Fosfomycin	3 (1.6%)	1 (0.7%)
Gentamicin	4 (2.1%)	0 (0.0%)
Levofloxacin	15 (8%)	8 (5.8%)
Linezolid	36 (19.3%)	4 (2.9%)
Meropenem	51 (27.3%)	8 (5.8%)
Metronidazole	2 (1.1%)	8 (5.8%)
Moxifloxacin	2 (1.1%)	0 (0.0%)
Piperacillin-tazobactam	35 (18.7%)	3 (2.2%)
Rifampin	2 (1.1%)	0 (0.0%)
Tedizolid	1 (0.5%)	0 (0.0%)
Teicoplanin	3 (1.6%)	2 (1.5%)
Tigecycline	2 (1.1%)	3 (2.2%)
Vancomycin	15 (8.0%)	3 (2.2%)
